# Classification of Trispanins: A Diverse Group of Proteins That Function in Membrane Synthesis and Transport Mechanisms

**DOI:** 10.3389/fcell.2019.00386

**Published:** 2020-01-22

**Authors:** Misty M. Attwood, Helgi B. Schiöth

**Affiliations:** ^1^Functional Pharmacology, Department of Neuroscience, Uppsala University, Uppsala, Sweden; ^2^Institute for Translational Medicine and Biotechnology, Sechenov First Moscow State Medical University, Moscow, Russia

**Keywords:** ionotropic glutamate receptor, membrane biosynthesis, cerebral cortex, fatty acid transport, lipid metabolic process, transmembrane proteins, trispanins

## Abstract

As the structure and functions of proteins are correlated, investigating groups of proteins with the same gross structure may provide important insights about their functional roles. Trispanins, proteins that contain three alpha-helical transmembrane (3TM) regions, have not been previously studied considering their transmembrane features. Our comprehensive identification and classification using bioinformatic methods describe 152 3TM proteins. These proteins are frequently involved in membrane biosynthesis and lipid biogenesis, protein trafficking, catabolic processes, and in particular signal transduction due to the large ionotropic glutamate receptor family. Proteins that localize to intracellular compartments are overrepresented in the dataset in comparison to the entire human transmembrane proteome, and nearly 45% localize specifically to the endoplasmic reticulum (ER). Furthermore, nearly 20% of the trispanins function in lipid metabolic processes and transport, which are also overrepresented. Nearly one-third of trispanins are identified as being targeted by drugs and/or being associated with diseases. A high number of 3TMs have unknown functions and based on this analysis we speculate on the functional involvement of uncharacterized trispanins in relationship to disease or important cellular activities. This first overall study of trispanins provides a unique analysis of a diverse group of membrane proteins.

## Introduction

Cellular boundaries or membranes are imperative for cells to function properly and membrane formation is considered an essential step in the emergence of life. The plasma membrane is the most studied of cell membranes, and is composed of a phospholipid bilayer that contains phospholipids, glycolipids, sterols, and proteins. In addition to the plasma membrane, eukaryotic cells also contain intracellular membrane-bound organelles, including double membrane compartments such as the nucleus and mitochondria and single membrane-bound organelles such as the endoplasmic reticulum (ER), Golgi apparatus, lysosomes, peroxisomes, and vesicles. Each membrane-bound compartment is defined by its lipid and protein composition (Harayama and Riezman, 2018). For instance, localized sphingolipid metabolism is associated with membrane budding and formation of exosomes in multivesicular bodies, or in another example, membrane proteins are differentially transported to the apical or basal plasma membrane in polarized cells (Harayama and Riezman, 2018). Membranes act as barriers that not only physically separate the intracellular components from the extracellular environment or from each other, but selectively allow materials to flow in and out of the cell or other organelles, as well as extract energy from the environment (Thomas and Rana, 2007). Transmembrane proteins and complexes can form channels, pores, and gates that extend through the lipid bilayer and function in active and passive transport of substrates across a membrane. Membrane machinery and protein complexes on different organelles can be involved in specialized activities, for example vesicle formation and movement on the Golgi apparatus, protein targeting and transport at the ER, and specific receptor signaling and transduction at the plasma membrane. And due to the varied essential functional pathways that membrane proteins are involved in, they are of particular interest in investigating the pathophysiology of diseases. In fact, membrane proteins are major targets for pharmaceutical agents and more than 60% of drug targets are membrane proteins (Overington et al., 2006).

Of the ∼20,000 total protein coding genes in *Homo sapiens*, approximately 25–30% (∼5500) are coded for transmembrane proteins (Almén et al., 2009; Attwood et al., 2017). Further, ∼70% of these membrane proteins are estimated to be conserved since the last holozoa common ancestor, indicating the fundamental importance and evolutionary origins of many membrane proteins (Attwood et al., 2017). Investigating cell membrane structures and complexes harkens back to how life originated, i.e., how the lipid bilayer developed and how metabolism and replication evolved in cells (Griffiths, 2007; Thomas and Rana, 2007; Deamer, 2017). Further, membrane compartmentalization and expansion enabled the development of larger cells and the separation of cell functions in eukaryotic cells, which also inaugurated the need for communication between the different cellular compartments (Gabaldón and Pittis, 2015). Subcellular compartmentalization has been aided through evolutionary retargeting of membrane proteins to shared or different localizations, which is evident by the varied internal sorting of proteins across different physiological conditions, cell types, localizations, and lineages (Gabaldón and Pittis, 2015). Consequently, investigating the evolutionary developments of homologous protein families as well as where proteins localize to can provide insight into their different functional activities.

The membrane proteome can be characterized into functional groups based on the number of transmembrane helices; i.e., the tertiary structure of protein groups can promote specific functional activities. A widely known example is the large seven transmembrane spanning families of G protein-coupled receptors (GPCRs) that function in signaling (Lagerström and Schiöth, 2008) or the twelve transmembrane proteins of the major facilitator superfamily (MFS) that act as transporters (Reddy et al., 2012). Additionally, studies have shown that proteins that span the membrane once or twice are often engaged in enzymatic functions (Almén et al., 2009) or immune response signaling (Sällman Almén et al., 2012) while many tetra-spanning proteins are involved in transport activities (Attwood et al., 2016). Our previous investigation has suggested that there are many three transmembrane-spanning (3TM or trispanins) proteins that have unknown functions (Almén et al., 2009) and an in-depth analysis of membrane proteins that share a basic structural similarity with 3TM regions has yet to be published. Investigations of groups of proteins with the same gross structure may lead to important insights about the functional activities, perhaps in particular for proteins with unknown functions.

In this study we perform comprehensive bioinformatic analyses of all 3TM proteins in the human genome. We identify and characterize the 3TM group characteristics and incorporate information on relevant functional activities along with cellular localizations, tissue enrichment patterns, and protein-protein interaction networks to describe the predominant functional activities of this group of proteins. Additionally, we use this methodology to speculate on the functions of several uncharacterized trispanins that are associated with disease or potentially involved in important pathways.

## Functional Analysis Results

We identified 152 proteins as 3TMs and these were classified into three primary functional classes plus a fourth group that has varied activities: 35 proteins were identified as enzymes that had an associated enzyme commission number (EC); 26 proteins had an associated transporter classification database identifier (TCDB) and were characterized as transporters; 21 receptors were identified based on IUPHARS guide to pharmacology descriptions; and 43 proteins with varied functional activities were characterized as well as 27 proteins that were classified as uncharacterized. Approximately one-third of trispanins localize to multiple membranes, with the plasma membrane and another intracellular organelle being the predominant combination. The original CCDS human protein sequences file contained 18,894 unique CCDS gene identifiers with 32,554 total entries including isoforms. The sequences were pre-processed and the signal peptides were excised, followed by evaluation with TOPCONS-single to predict transmembrane helices. The 3TM dataset was collated by removing ambiguous entries, isoforms, and manually adding proteins ascertained as 3TM through literature searches (see section “Materials and Methods” for details). Functional annotations and localizations were assessed through gene ontology (GO) descriptions (Ashburner et al., 2000; The Gene Ontology Consortium, 2019), the human protein atlas (Uhlén et al., 2015), the PANTHER classification database (Mi et al., 2019), and KEGG pathways database (Kanehisa et al., 2017). Supplementary File S1 contains the final 3TM dataset.

### Membrane Complexes

**FIGURE 1:**
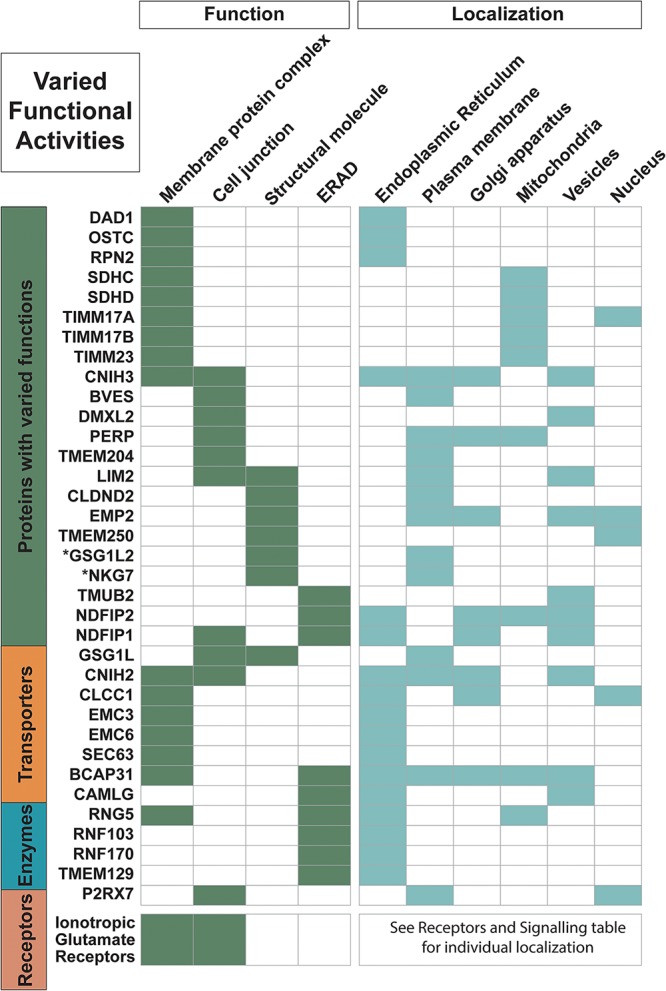
Proteins with varied functional activities and localization information. Trispanins from all four major classes involved in these common functional activities: membrane protein complexes; cell junctions; structural molecules; and endoplasmic reticulum (ER)-associated degradation processes (ERAD) and ubiquitination processes. All 18 ionotropic glutamate receptors are included in one row and individual localization information on them can be found in Figure 4. Functional activities and localization descriptions derived from gene ontology (GO) Annotations (Huntley et al., 2015), PANTHER classifications (Mi et al., 2019), and the human protein atlas (Uhlén et al., 2015).

Almost 35 of the proteins in the dataset are members of membrane complexes and localize to various membranes, including the plasma membrane, as well as organelles such as the mitochondrion, the endomembrane system with the ER and Golgi apparatus, and the nucleus (Figure 1). With the 17 proteins that localize to the mitochondrion, five of the nine proteins that are found in the inner mitochondrial membrane are involved in inner mitochondrial membrane complexes, including three members (TIMM23, TIMM17A, and TIMM17B) of the TIM23 translocase. The TIM23 complex is the major translocase for importing proteins across the inner mitochondrial membrane and into the mitochondrial matrix (Demishtein-Zohary and Azem, 2017). SDHC and SDHD are also members of inner mitochondrial membrane complexes. They are the two transmembrane components of succinate dehydrogenase, or the mitochondrial respiratory complex II, which functions in the mitochondrial electron transport chain, and these trispanins together contain one heme *b* and provide the binding site for ubiquinone (Yankovskaya et al., 2003). The trispanin TMEM177 has been recently discovered to form complexes with COX20 and associate with COX2, which are essential for the assembly of cytochrome c oxidase that is the final enzyme complex of the mitochondrial respiratory electron transport chain. TMEM177 dynamically interacts with COX2 subcomplexes in a COX20-dependant manner which in turn stabilizes COX2 during early synthesis (Lorenzi et al., 2018). TMEM11 is another inner mitochondrial protein and is associated with the multiple mitochondrial contact site and cristae junction organizing system (MICOS) complex, which dynamically regulates mitochondrial membrane architecture (Guarani et al., 2015). Both TMEM177 and TMEM11 have yet to be annotated as members of membrane protein complexes, which may contribute to an underrepresentation of complexes in the dataset.

Several membrane complexes that localize to the ER function in two different pathways that facilitate membrane insertion of proteins. One pathway uses the important signal recognition particle (SRP) dependent ER protein translocon, which consists of the co-translational protein-conducting channel Sec61 complex along with additional subunits involved in nascent chain processing and translocation (Mades et al., 2012). The SEC63 protein identified in the dataset is part of the Sec61 chaperone network that performs substrate-selective quantity control during co-translational ER import (Mades et al., 2012). An additional three proteins in the dataset are members of the oligosaccharyl-transferase (OST) complex: RPN2, DAD1, and OSTC (also known as DC2). The OST complex is also an integral component of the translocon that catalyzes co-translational N-glycosylation, which is one of the most common protein modifications in eukaryotic cells (Pfeffer et al., 2014; Braunger et al., 2018). Furthermore, homologs of BCAP29 and BCAP31, both members of the 3TM dataset, have been shown to form a complex together that is associated with the Sec61 complex and interacts with translocation substrates (Wilson and Barlowe, 2010). Two more proteins, WRB and CAML, are involved in another type of protein insertion into the ER membrane as subunits in the post-translational tail-anchored membrane protein insertion TRC40 complex (Yamamoto and Sakisaka, 2012).

Moreover, nearly 45% of the proteins in the dataset localize to the ER and function in activities such as protein biogenesis, folding, sorting, trafficking, and degradation. At least nine proteins are identified in the ER-associated protein degradation (ERAD) pathway and ubiquitination system. ERAD monitors the biogenesis and folding of membrane and secretory proteins in the ER and targets misfolded proteins for ubiquitination and subsequent degradation (Ruggiano et al., 2014). And at least two members of the ER membrane protein complex (EMC3 and EMC6), which is engaged in protein folding, are identified in the dataset. Additionally, several proteins are identified in ER morphogenesis and tubular organization network.

More than 70 proteins are described with engaging in various protein-protein interactions, with nearly half of them (33 proteins) annotated as forming homo- and/or heteromeric subunits of complexes. All 18 of the ionotropic glutamate receptors are identified in this group, which is expected as the structural arrangements of the subunit pairings, the ligand binding domains, as well as the N-terminal domains are the focus of intense research and several conformations have been experimentally determined; for review see Green and Nayeem (2015). Furthermore, several members of protein families in the dataset form heteromeric subunits including BCAP29 and BCAP31, NDFIP1, and NDFIP2 as well as other proteins that self-associate to form homomeric units such as BVES, MGST2, TMEM109, TMEM18, SGPL1, STS, SLC27A1, and SLC31A1.

### Transport and Trafficking

**FIGURE 2:**
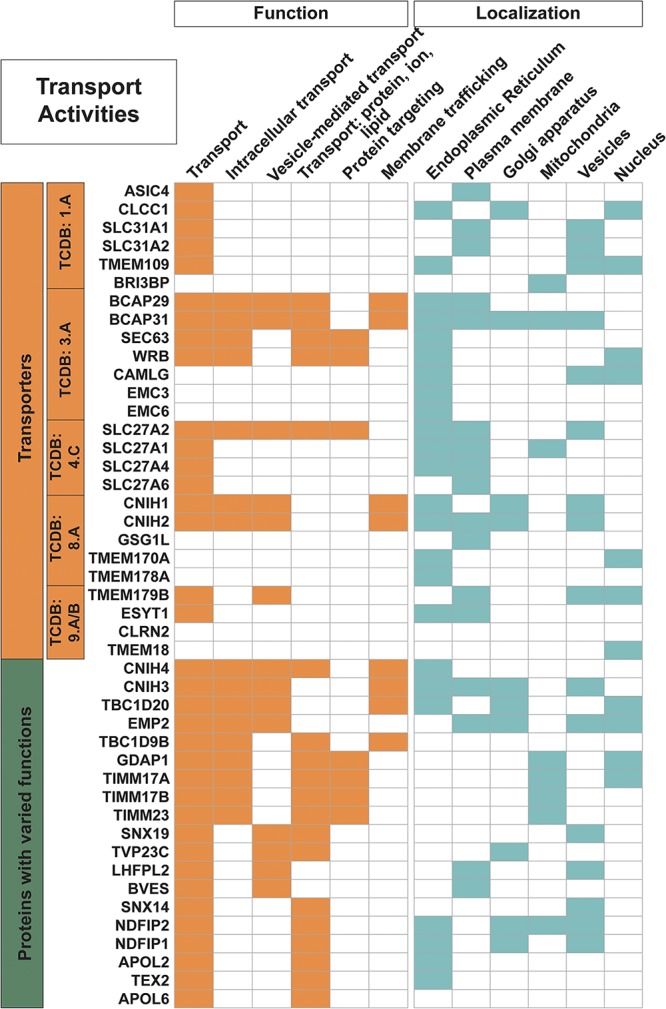
Trispanins involved in transport activities. All 26 proteins identified with a transporter classification database (TCDB) identifier (descriptive boxes on left) are included in this figure. Only 40 of the 61 total trispanins involved in transport activities are shown; however, all proteins are shown that are involved in intracellular transport; vesicle-mediated transport; transport of proteins, ions, and lipids; protein targeting; and membrane trafficking. The four members of the SLC27A1/2/4/6 also contain an enzyme commission number (EC) and are shown in Figure 3. Functional activities and localization descriptions derived from gene ontology (GO) Annotations (Huntley et al., 2015), PANTHER classifications (Mi et al., 2019), and the human protein atlas (Uhlén et al., 2015).

Overall, 40% of trispanins are engaged in transport activities, with nearly 20% involved in intracellular transport and protein trafficking as well as membrane and vesicle trafficking (Figure 2). The majority of the trispanin transport activity is the movement of cargo, for example proteins and other macromolecules, around the cell utilizing small compartments of membranes called transport vesicles. Nine proteins participate in membrane trafficking and include the four members of the cornichon family. The cornichon (CNIH1-4) family members function as cargo receptors in ER export and are involved in the selective transport of TGF-alpha family proteins in COPII vesicles (Zhang and Schekman, 2016), while CNIH4 also acts as a cargo-sorting receptor that recruits GPCRs into COPII vesicles for export from the ER to the cell surface (Sauvageau et al., 2014). Additionally, CNIH2 and CNIH3 co-assemble as auxiliary subunits of AMPA receptors where they increase surface expression of AMPARs and alter channel gating (Schwenk et al., 2009). TBC1D20 is another predicted trispanin involved in COPII-coated vesicle cargo loading and Rab GTPase activating activity (Haas et al., 2007).

Nearly 30 proteins localize to vesicles including secretory vesicles (8 proteins), lysosomes (4), endosomes (8), exosomes (5), and Golgi-associated vesicles (4) to engage in a variety of activities. Furthermore, the dataset also contains proteins involved in vesicle formation and regulation, such as RAB5IF and EMC6, which both contain the Rab5 interacting protein Pfam domain (Rab5ip; PF07019). RAB5IF may function on endocytic vesicles as a receptor for rab5-GDP and be involved in the activation of RAB5A, which is an important regulator of the endocytic pathway (Hoffenberg et al., 2000), while EMC6 has been reported to interact with RAB5A and BECN1 and regulate autophagosome formation (Li et al., 2013). TBC1D9, also in the 3TM dataset, may regulate the membrane trafficking pathway to the late-endosome and/or Golgi apparatus in spermatocytes and in spermatogenesis (Nakamura et al., 2015). Another protein family with two mammalian homologs, the Nedd4 Family-interacting Proteins 1 and 2 (NDFIP1-2), is also involved in orchestrating protein trafficking, which may contribute to why they both localize to multiple regions, including the ER, Golgi apparatus and vesicles and NDFIP2 also localizes to the mitochondria. Both proteins bind to and activate members of the Nedd4 family of E3 ubiquitin ligases, which in turn targets proteins for degradation by the proteasome (Putz et al., 2008). NDFIP1-2 are important for protein trafficking via exosomes, which originate from late endosomes and multivesicular bodies (MVB) and provide a rapid manner of discarding unwanted proteins as well as functioning in cell to cell communication (Putz et al., 2008).

The 26 transporter proteins with an associated TCDB identifier include a variety of transporter classes. Six proteins are identified as alpha-type channels/pores (TCDB:1.A). This includes the important SLC31 family of copper transporters, which are involved in the maintenance of copper homeostasis and regulate intracellular copper concentration (Schweigel-Röntgen, 2014). Seven proteins are involved in transport systems that hydrolyze the diphosphate bond of inorganic pyrophosphate, ATP, or another nucleoside triphosphate to drive the active uptake or extrusion of a solute(s) (TCDB:3.A). Five of these proteins are involved in complexes of the previously mentioned Sec61 ER translocon. Four proteins are members of the acyl CoA ligase-coupled transporters (TCDB:4.C), which are group translocators that modify the transported substrate during the transport process. These four SLC27A proteins also engage in enzymatic activities; however, they are classified as transporters in this dataset to remove redundancy. Five proteins are identified as accessory factors involved in transport (TCDB:8.A) and four proteins are transporters of unknown classification (TCDB:9.A and 9.B). It is interesting to note that contrary to the many transporters that form oligomeric subunits to transport molecules across membranes (Alguel et al., 2016), only seven of the proteins identified in the dataset with a TCDB number are annotated to form homo- or heteromeric units. Many of the identified trispanin transporters appear to be engaged in other transport activity, for example intracellular transport, vesicle-mediated transport, and protein targeting.

### Lipid Biogenesis and Metabolic Processes

**FIGURE 3:**
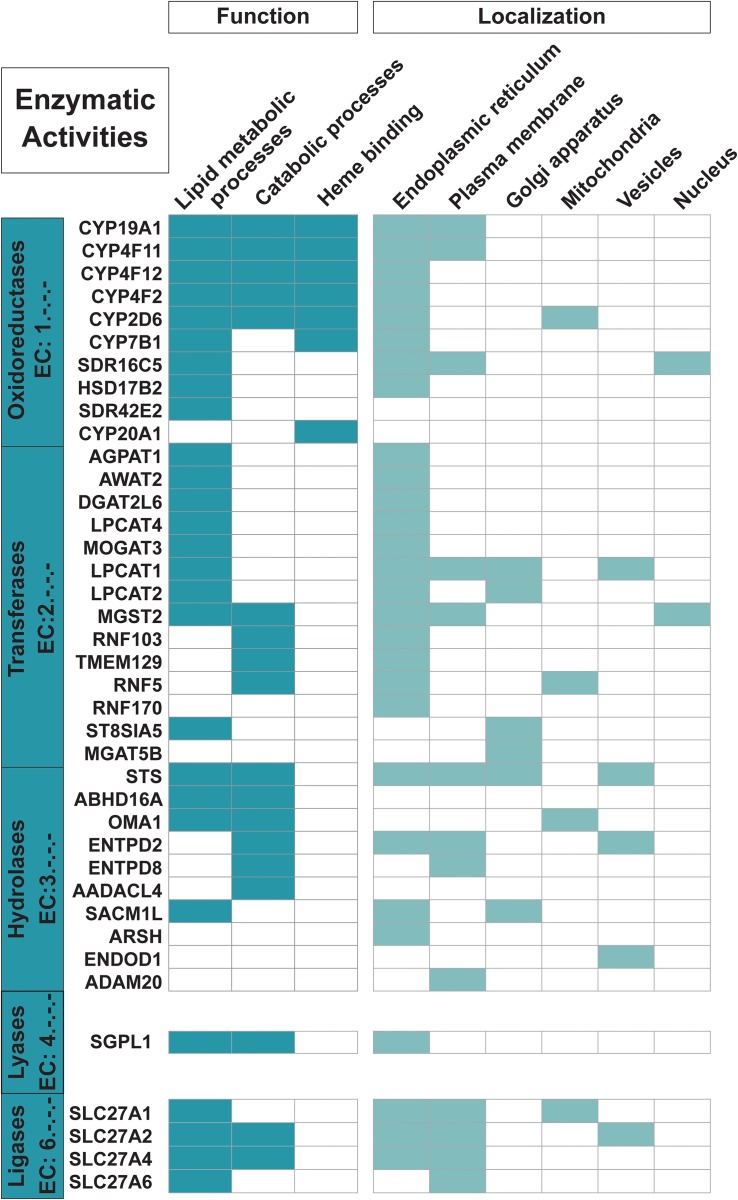
Proteins with enzymatic functions. The 35 trispanins with an associated Enzyme Commission (EC) number (descriptive boxes on left) are shown along with the predominant functional activities including lipid metabolic processes, catabolic processes, and heme binding. Only 22 of the 31 trispanins involved in lipid metabolic processes and 16 of the 24 total proteins active in catabolic processes are shown here. The four members of the SLC27A1/2/4/6 are also classified as transporters. Functional activities and localization descriptions derived from gene ontology (GO) annotations (Huntley et al., 2015), PANTHER classifications (Mi et al., 2019), and the human protein atlas (Uhlén et al., 2015).

Nearly 20% of the proteins in the dataset are involved in lipid metabolic processing and lipid transport (Figure 3). And as expected, nearly all of these proteins localize to the ER which is the main site for lipid synthesis. Four members of the 1-acylglycerol-3-phosphate O-acyltransferases (AGPAT) gene family (AGPAT1, LPCAT1, LPCAT2, and LPCAT4) are predicted to be trispanins. These enzymes are involved in maintaining the composition of fatty acyl chains that make up phospholipids, which are the major constituents of membranes (Wang and Tontonoz, 2019). Four members out of six of the SLC27A family of fatty acid transporters were predicted to contain three transmembrane helices (SLC27A1, SLC27A2, SLC27A4, and SLC27A6). The two other members of the family had conflicting transmembrane predictions from none to two to six and were not included in the dataset. This family is unusual in that their multifunctional activities include both transport and enzymatic activation of long chain fatty acids, which can then be used by the cell in many metabolic processes including phospholipid synthesis (Anderson and Stahl, 2013). Seven enzymes in the dataset are members of the Cytochrome P450 family, including four enzymes (CYP4F2, CYP4F11, CYP4F12, and CYP4B1) that play primary roles in the omega-hydroxylation of endogenous long chain and very long chain fatty acids, which include the physiologically important eicosanoids, prostaglandins, leukotrienes, and arachidonic acid (Johnson et al., 2015). Furthermore, CYP4F2 is primarily responsible for the formation of the important signaling metabolite 20-HETE. Catabolic processes are other functional activities trispanins are involved in, with approximately 16% (24 proteins) engaged in the dataset and 16 enzymes involved (shown in Figure 3). These proteins are involved in chemical reactions and pathways that result in the breakdown of substances including the apoptotic signaling pathway, alkaloid catabolic processes, steroid catabolic processes, and leukotriene B4 catabolic processes.

### Signal Transduction

**FIGURE 4:**
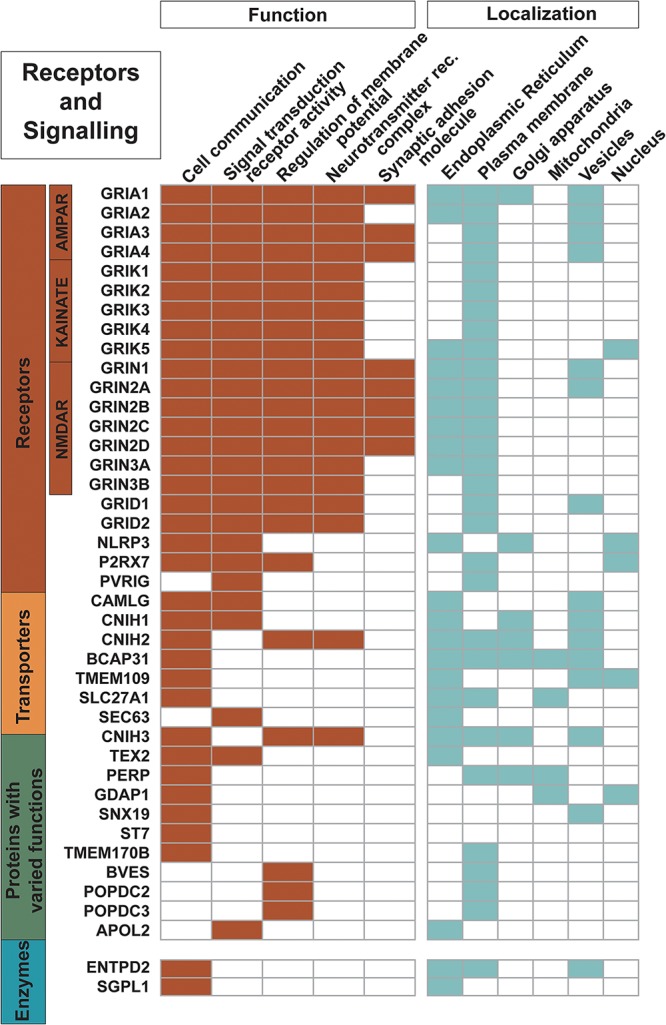
Proteins engaged as receptors and in signaling. The 21 receptors in the dataset along with other trispanins involved in cell communication; signal transduction receptor activity; regulation of membrane potential; neurotransmitter receptor complexes; and synaptic adhesion molecules are shown here. The 18 ionotropic glutamate receptors are heavily involved in these activities. Functional activities and localization descriptions derived from gene ontology (GO) annotations (Huntley et al., 2015), PANTHER classifications (Mi et al., 2019), and the human protein atlas (Uhlén et al., 2015).

Twenty-one proteins are identified as receptors while an additional five more proteins are involved in signal transduction activity (Figure 4). The 18 proteins of the ionotropic glutamate receptor family are characterized as trispanins. The proteins in this receptor family contain three transmembrane helices with a fourth central pore-like helix that does not fully span the membrane. Hence, membrane prediction software can give conflicting results with this family and the X-ray resolved structure for an AMPA-subtype glutamate receptor was referenced to include all members (Sobolevsky et al., 2009). The ionotropic glutamate receptor family is grouped into four different classes based on pharmacology and structural homology and include: the four AMPA receptors (GRIA1-4); the five kainate receptors (GRIK1-5); the seven NMDA receptors (GRIN1, GRIN2A-D, and GRIN3A-B); and two delta receptors (GRID1-2). Glutamate receptor subunits co-assemble to form ligand-gated ion channels that mediate fast excitatory synaptic transmission in the central nervous system and regulate a broad spectrum of processes in the brain, spinal cord, retina, and peripheral nervous system (Traynelis et al., 2010). As such, a significant volume of literature has been published regarding ionotropic glutamate receptors and will not be covered here; for example, see Traynelis et al. (2010); Reiner and Levitz (2018) for review. The three additional proteins identified as receptors in the dataset are: NACHT, LRR and PYD domains-containing protein 3 (NLRP3); P2X purinoceptor 7 (P2RX7); and Transmembrane protein PVRIG (PVRIG). As expected, virtually all of the receptors (except PVRIG) are annotated as involved in cell communication; moreover an additional 17 trispanins are also engaged cell communication such as Wnt signaling, insulin secretion, adiponectin-activated signaling, apoptotic signaling, and calcium-mediated signaling.

### Structural Support Activity

Seven proteins are identified as cytoskeletal proteins that provide structural support according to GO annotations and PANTHER protein class analysis (Figure 1). Four proteins (LIM2, NKG7, CLDND2, and EMP2) contain the PMP-22/EMP/MP20/Claudin family (PF00822) Pfam domain, whose members perform diverse functions. Proteins containing this domain are often predicted to have four transmembrane helices; however, these four more distantly related proteins to mammalian claudins were predicted to have 3TMs. The first N-terminal helix was identified to be a signal peptide that was detected with the recently released SignalP v5.0 software (Armenteros et al., 2019b). The 3TM predictions for these proteins are also in agreement with the membrane predictions of the human protein atlas data, which uses a majority decision method to determine presence of signal peptides and transmembrane regions (Fagerberg et al., 2010; Uhlén et al., 2015).

### Membrane Topology and Signal Peptide Predictions

The orientation of the N- and C-termini in relation to the cytoplasmic side or non-cytoplasmic side (i.e., extracellular region or organelle lumen) can indicate functional activities proteins are engaged in. For example, GPCRs have extracellular N-termini that interact with cognate ligands and function in signal transduction (Coleman et al., 2017). However, the connection between function and membrane topology was difficult to distinguish with most of the trispanins as 72 of the trispanins had the N-terminus predicted in the cytoplasmic region (in) and 80 proteins had the N-terminus in a non-cytoplasmic environment (out). Receptors were the only group that had a skewed proportion with one protein with the N-terminus in the cytoplasmic environment while 20 proteins were non-cytoplasmic. The N-terminus of the 18 ionotropic glutamate receptors were all categorized as non-cytoplasmic (out) in accordance with the experimentally determined GRIA2 amino-terminal domain (Jin et al., 2009). Enzymes had 18 proteins with the N-terminus predicted in with 17 out; transporters had 11 in and 15 out; proteins with varied functional activities had 25 in and 18 out; and uncharacterized trispanins had 17 in and 10 out.

N-terminus signal peptides are short peptides that direct proteins to different locations in the cell including the ER, plasma membrane as well as other intracellular organelle membranes. Using multiple localization prediction resources (see section “Materials and Methods” for details), 50 N-terminal signal peptides were predicted in trispanins; 44 of these proteins have associated annotations that they localize to the ER, plasma membrane, mitochondria, and/or Golgi apparatus. Moreover, C-terminus signaling sequences as well as internal signal retention sequences are also methods of directing proteins to the ER (Ouzzine et al., 1999). Interestingly, in addition to the 64 trispanins that are already annotated as ER resident proteins, 53 more proteins in the dataset are predicted to localize to the ER and 17 are predicted to localize to the plasma membrane that do not have any associated annotation. Three additional trispanins were predicted to localize to the mitochondria and two more proteins were predicted to travel to the Golgi apparatus. One limitation with various localization resources seems to be that the results only give one localization area, while experimental evidence gives multiple locales the protein is found.

### Disease Involvement and Drugs Targeting 3TMS

**FIGURE 5:**
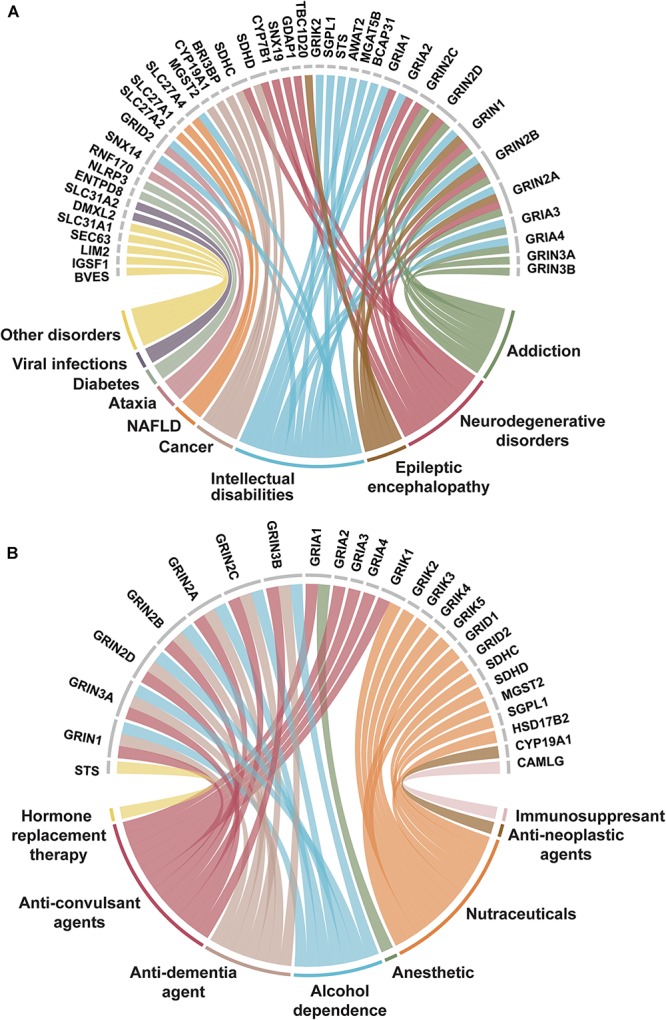
Trispanins identified in disease-gene associations and clinically established by FDA approved drugs. **(A)** Disease-gene associations with trispanins. The 41 proteins identified with disease-gene associations are shown at the top of the figure while the corresponding diseases are linked through color and identified in the bottom part. The disorders include: intellectual disability; addiction; neurodegenerative diseases including Alzheimer’s disease, amyotrophic lateral sclerosis, Huntington’s disease, and Parkinson’s disease; epileptic encephalopathy; cancer; non-alcoholic fatty liver disease (NAFLD); ataxia; diabetes mellitus; viral infections; and other. The disease-gene association data were collated from Jensen Diseases resource (Pletscher-Frankild et al., 2014) and DisGenet database (Piñero et al., 2017). **(B)** Trispanins targeted by FDA approved drugs. The 26 clinically established trispanins that have been targeted by FDA approved drugs are shown. The established drug targets are presented at the top of the figure and the corresponding therapeutic category of drugs that target each protein is color-linked and described on the bottom half. The FDA approved drug-target information comes from an updated dataset that provides curated information on FDA approved drug targets (Rask-Andersen et al., 2014; Attwood et al., 2018) as well as DrugBank (Wishart et al., 2017). Trispanins that were targeted and mediated the therapeutic action are included, while proteins as secondary targets or unknown mechanisms of action were not included. FDA: US Federal Drug Administration.

Nearly one-third (48 proteins) of the dataset was identified as being targeted by drugs and/or being associated with diseases. Disease-gene associations with the trispanins were particularly identified with neurological disorders including epilepsy, Alzheimer’s disease, amyotrophic lateral sclerosis, Huntington’s disease, Parkinson’s disease, and also mental or behavioral disorders such as substance dependence and intellectual disabilities (Figure 5A). The emphasis on neurodegenerative disorders is expected due to the important ionotropic glutamate receptor family and their involvement in the underlying pathophysiology of many disorders (Reiner and Levitz, 2018). Disease-gene associations with disorders of the eye, cancer, infectious viral diseases like pertussis, human papilloma virus, and hepatitis were also identified in the dataset.

Of the 26 trispanins that are targeted by federal drug administration (FDA) approved drugs, approximately 70% of them are members of the ionotropic glutamate receptor family (Figure 5B). The FDA approved drugs that have established these trispanins as validated targets are therapeutically categorized as anesthetics, anti-neoplastic agents, anti-convulsants, nutraceuticals, and also anti-dementia agents for different conditions. The proteins in the dataset have also been targeted by immunosuppressive, and anti-addiction agents. The 3TM proteins have also been targeted by investigative drugs in clinical trials for such conditions as sleep disorders, depression, cardiovascular diseases, tinnitus, and Alzheimer’s disease. Membrane proteins have always been primary targets for pharmaceutical agents due to the critical functions they are involved with and the wide-reaching signaling pathways they participate in. And in fact, with the continued technological advancements in biopharmaceutical engineering, multipass membrane proteins are expected to be of increasing interest as antibody targets (Carter and Lazar, 2018).

## Discussion

**FIGURE 6:**
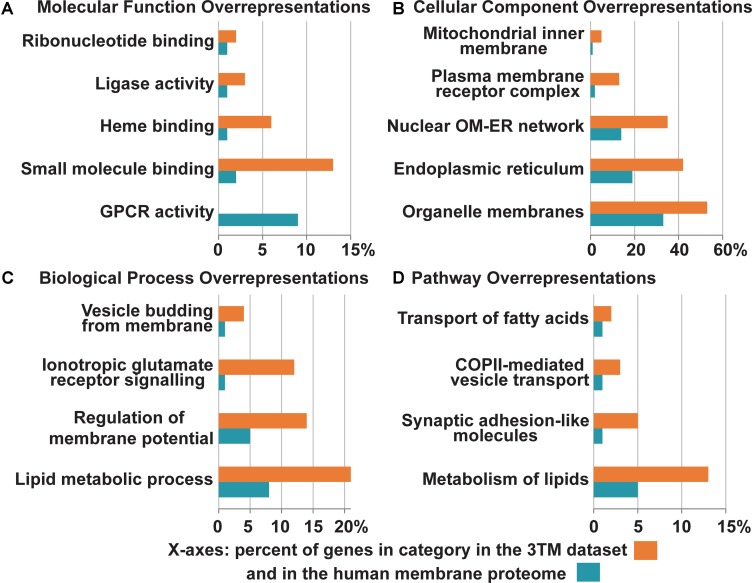
Overrepresentation of select Gene Ontology terms identified in the 3TM dataset in comparison to the entire human transmembrane proteome. The *x*-axes are the percent of genes in each category identified in the 3TM dataset (orange) in comparison to the percent of genes in the human transmembrane proteome (blue). Select pathway overrepresentations from the PANTHER Overrepresentation Test are presented in all four sections (see Supplementary Table S1 for full statistical information). **(A)** Molecular pathways. As the glutamate-gated ion channel protein family is an important receptor/transporter family involved in many activities, specific pathways were chosen to highlight the breadth of different activities that trispanins are overrepresented in. Twenty proteins are involved in small molecule binding, which are predominantly the ionotropic glutamate receptor families. Nine enzymes including the seven members of the cytochrome p450 family identified in the dataset engage in heme binding. However, one area that the 3TM dataset is significantly underrepresented in is in GPCR activity. **(B)** Cellular localizations. The 3TM dataset is rich in proteins that localize to organelle membranes, with the ER in particular and also the nuclear outer membrane-ER network. Trispanins that localize to the mitochondrial inner membrane are also overrepresented due to the proteins functioning in the mitochondrial import inner membrane translocase complex. The ionotropic glutamate receptor families compose the overrepresented group of plasma membrane receptor complexes. **(C)** Biological pathways. More than 30 trispanins are involved in lipid metabolic processes, including those involved in lipid synthesis and remodeling, activation of long chain fatty acids, and membrane genesis. Six proteins are involved with membrane trafficking and vesicle budding from the membrane. **(D)** Reactome pathway. Several pathways were found to be overrepresented in the Reactome pathways dataset (v65, released on 2019-03-12). The NMDAR and AMPAR glutamate families interact with synaptic adhesion-like proteins which function in protein-protein interactions at synapses in the neuronal system. Many of the proteins involved in the metabolism of lipids function in lipid biosynthesis, mobilization, transport, and activation. Several of the members of the SLC27A family function specifically in the transport of fatty acids. The proteins involved in vesicle-mediated transport via COPII components traffic cargo from the ER to the ER-Golgi intermediate compartment. The overrepresentation analysis is from the PANTHER (Mi et al., 2019) overrepresentation test (v14.1) with the gene ontology (GO) Annotation (Huntley et al., 2015) database released on 2019-07-03. Fisher’s Exact test was performed and the False Discovery Rate was calculated with *p* < 0.05. The human membrane protein identities is from Attwood et al. (2017) with 5723 of 5777 proteins and 150 of 152 3TM proteins successfully mapped using GO annotation. GPCR, G protein-coupled receptor; OM, outer membrane; ER, endoplasmic reticulum.

**FIGURE 7:**
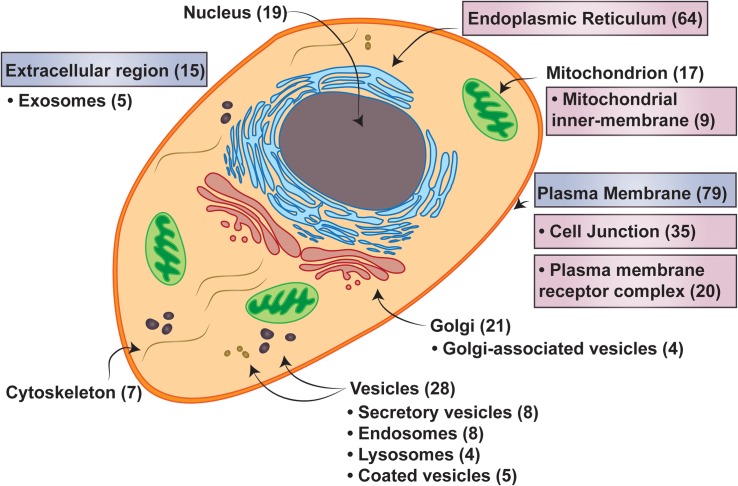
Cell localizations of three transmembrane spanning proteins. Major cellular localizations presented with the number of proteins identified in the 3TM dataset in parenthesis. Labels in boxes with pink hues are identified as statistically overrepresented, while the boxes with the blue hues are underrepresented in the 3TM dataset in comparison to the entire human membrane proteome (see Supplementary Table S1 for full statistical information). Localization data is extracted from QuickGO annotation web-based tool (Binns et al., 2009). The overrepresentation analysis is from the PANTHER (Mi et al., 2019) overrepresentation test (v14.1) with the gene ontology (GO) Annotation (Huntley et al., 2015) database released on 2019-07-03. Fisher’s Exact test was performed and the false discovery rate was calculated with *p* < 0.05. The human membrane protein identities is from Attwood et al. (2017) with 5723 of 5777 proteins successfully mapped and 150 of 152 trispanins successfully mapped using GO annotation.

Many of the proteins that comprise the 3TM dataset function in aspects of cellular membrane remodeling and trafficking systems including membrane synthesis, lipid metabolism, protein trafficking, membrane imbedded complexes, and also ionotropic glutamate receptor signaling (Figure 6). Proteins that localize to organelle membranes are overrepresented in comparison to the entire *Homo sapiens* membrane proteome [Fold Enrichment (FE) = 1.57; FDR = 3.00e-04), and in particular the ER (FE = 2.15; FDR = 2.29e-07) and nuclear outer membrane- ER membrane network (FE = 2.40; FDR = 4.06e-07; all *p*-values < 0.05)] (Figure 7). This is consistent with the overrepresentation of proteins involved in fatty acid and lipid metabolic processes (FE = 6.15; FDR = 5.13e-04 and FE = 2.25; FDR = 1.41e-02, respectively) as the ER and Golgi apparatus are major sites of *de novo* membrane lipid synthesis and also hubs for directing protein trafficking (Fagone and Jackowski, 2009). Conjointly, the 3TM dataset contains a spectrum of proteins that are involved in membrane genesis through lipid biosynthesis and remodeling. Specifically, 3TM members of the SLC27A family uptake, transport and also have the unique ability to activate long chain fatty acids (Anderson and Stahl, 2013) while members of the AGPAT family are responsible for catalyzing fatty acid transfer between acyl donor and acceptor and the incorporation of fatty acids into various lipids such as phospholipids (Yamashita et al., 2014). Furthermore, the compositions of phospholipids are regulated after *de novo* synthesis in a process called fatty acid remodeling, and the dataset contains acyltransferases that are involved in both *de novo* synthesis as well as the important remodeling activities. The varied phospholipids that result from the remodeling process produce compositional membrane diversity, which is necessary for membrane fluidity and curvature (Hishikawa et al., 2008). Moreover, at least two of the AGPAT enzymes in the dataset function in phospholipid remodeling in the Lands’ cycle to produce phosphatidylcholine (PC) (Hishikawa et al., 2008), which is the most abundant glycerophospholipid in mammalian cell membranes – comprising ∼40–50% of total phospholipids (Wang and Tontonoz, 2019). Hence, with 20% of the proteins in the dataset involved in lipid metabolic processes and lipid transport, these are key activities for 3TMs.

Another interesting aspect of the 3TM dataset is that more than 20% of the proteins act as subunits of membrane-imbedded complexes, such as the TIMM23 translocase complex, OST complex, and the ER membrane complex. Proteins involved in both co-translational SRP dependent membrane insertion complexes as well as post-translational tail-anchored membrane insertion complexes are represented in the dataset. Moreover, proteins ensuring proper folding with the ER membrane complex as well as clearing misfolded proteins through the ERAD process are also classified in the dataset. Further literature investigation showed that GO annotation and PANTHER classifications have not yet included all relevant annotations for the 3TM dataset, and hence the overrepresentation analyses may yield conservative results in comparison to the actual functional activities the proteins are involved in. For example, BCAP29 and BCAP31 appear to form a complex that associates with the ER translocation apparatus and interacts with translation substrates (Wilson and Barlowe, 2010), but there is not associated GO annotations regarding these functions yet.

It is notable that proteins identified as receptors are under-represented in the 3TM dataset, with 13% identified in comparison to ∼25% in the entire *Homo sapiens* membrane proteome. However, the percent of enzymes (22%) and transporters (16%) are roughly comparable to the membrane proteome, which has 20% and 17%, respectively. As all eighteen members of the ionotropic glutamate receptor family are included in the 3TM dataset and they are involved in several unique essential activities, many of the functions they are involved in are overrepresented in the PANTHER analysis. Glutamate receptors play vital roles in the mediation of excitatory synaptic transmission in which neurons communicate with each other. Hence, for example, neurotransmitter receptor activity and regulation of postsynaptic membrane potential are overrepresented in the analysis (FE = 6.11; FDR = 1.73e-06 and FE = 7.51; FDR = 5.90e-06, respectively). However, aside from this glutamate receptor family, the 3TM dataset is not heavily involved in direct receptor signaling, although they may act as accessory proteins in receptor complexes such as with CNIH2 and CNIH3.

### Tissue Expression and Enrichment

The TissueEnrich analysis demonstrated that almost 22% of the proteins in the 3TM dataset had enriched or enhanced expression in the cerebral cortex in comparison to the membrane proteome of *Homo sapiens*, and importantly that seven of these 37 proteins are uncharacterized. While it might be expected that protein expression might be overrepresented in the brain due to the large ionotropic glutamate receptor family, it is surprising that nearly 20% of the proteins specifically expressed in the brain are completely uncharacterized. Additionally, the testis had the second most abundant number of tissue specific genes expressed there, although they were not significantly overexpressed in comparison to the membrane proteome. Previous analysis has shown that more than 80% of all human proteins are expressed in the testis (Uhlén et al., 2015), so perhaps the high number of testis expressed genes is not unusual. However, three of the eleven proteins are uncharacterized, lending further curiosity into the nature of the not yet classified proteins into the dataset.

### Uncharacterized Proteins and Proposed Functional Activities

Roughly 15% of the proteins in the dataset are uncharacterized, yet several interact with other 3TM proteins and, in particular, important membrane complexes. Furthermore, they are localized to specific subcellular locations, such as the ER, and expressed in specific tissues, including the cerebral cortex, testis, and spleen (Table 1). Hence, using bioinformatic analyses and literature research, we can speculate on the activities these uncharacterized proteins are engaged in. For example, we might suspect that FAM8A1 is involved in ER protein folding or targeting due to its localization in the Golgi apparatus, expression in many tissues, and interactions with the fellow 3TM protein EMC3, which is engaged in protein folding, and also HERPUD1, which is involved with ERAD. Recently, it was shown that FAM8A1 has a central role as a cofactor with HERPUD1 in the assembly and activity of the Hrd1 complex, which is engaged in ERAD of aberrant proteins (Schulz et al., 2017). Interestingly, researchers previously determined that FAM8A1, which is inserted within a human endogenous retrovirus, has ubiquitous mRNA expression and a testis-specific transcript present in the haploid phase of spermatogenesis (Jamain et al., 2001). TMCO4 is another uncharacterized protein we might infer is involved in protein targeting or trafficking as it localizes to the ER, is expressed at varying levels in many tissues, and has been annotated from experimental data to interact with VPS29, which is a vacuolar sorting protein that prevents missorting of transmembrane proteins into the lysosomal degradation pathway.

**TABLE 1:** Selected uncharacterized proteins with interacting proteins.

**Uncharacterized**	**Protein atlas**	**Tissue expression**	**Interacting**	**Function of**	**Location of**
**3 TM protein**	**Localization**	**or enrichment**	**proteins**	**interacting proteins**	**interacting proteins**
FAM8A1	Golgi	Expressed-in-all	***EMC3****°*	***ER membrane complex – protein folding***	***ER/cytosol/microtubules***
			HERPUD1*	ER-associated degradation	ER
			SLC51A*	Transport	ER/PM
TMEM235	ER/PM	Cerebral cortex (Enriched)	MAG°	Adhesion molecule	PM
			OPALIN°	Myelin paranodal loops in CNS	PM
			***TMEM151A****°*	–	***Nucleoli/cytosol***
			***TMEM151B****°*	–	–
TMEM151B	–	Cerebral cortex (Enriched)	***TMEM235****°*	–	***ER/PM***
TMEM151A	Nucleoli/cytosol	Cerebral cortex (Enriched)	***TMEM235****°*	–	***ER/PM***
TMEM178B	Vesicles	Cerebral cortex (Enhanced)	TRAF2^!^	Protein ubiquitination pathway	Cytosol
			TNIP2^!^	Apoptosis, Transcription	Cytosol/nucleoplasm
TMEM244	–	Cerebral cortex (Enhanced)	Homologs interact with: SPX°	Long chain fatty acid import into cell	Secreted
			ARL13A°	GTP binding	–
			GABRB1°	GABA-A receptor	Intracellular
GSG1L2	–	Cerebral cortex testis (Enriched)	–	(Conserved Pfam domain GSG1-like protein: PF07803)	–
TMEM262	–	Testis (Enriched)	CD3G*	T cell receptor complex; clathrin-coated vesicle membrane	PM
TMEM239	–	Testis (Enriched)	FGD2^∧^	Guanine-nucleotide releasing factor	PM/cytoskeleton
			USP5^∧^	Ubiquitin conjugation pathway	Cytosol/nucleoplasm
			S100B^∧^	Regulation of protein phosphorylation in brain	Vesicles/nucleus
TMEM114	–	Seminal vesicle (Enriched)	***LIM2****^‡^*	***Eye lens protein***	–
			CHMP4B^‡^	MVB and endosomal sorting	–
TMEM218	PM/cytosol	Expressed-in-all	***GDAP1****^!^*	***Protein targeting***	***Cytosol/mitochondria***
TMCO4	ER	Mixed	VPS29*	Vacuolar protein sorting protein	Cytosol/vesicles
TMEM220	Nucleoplasm	Expressed-in-all	COX6B1*	Cytochrome-c oxidase activity	Mitochondria
			SRC*	Non-receptor tyrosine kinase	PM/cytosol
C6ORF47	Cytosol	Mixed	FGFR3*	Tyrosine-protein kinase	ER
TMEM222	PM/cytosol	Expressed-in-all	ABHD16A*	***Lipid metabolic process***	–
C16ORF58	Intracellular membranes	Expressed-in-all	IFNGR1*	Interferon-gamma receptor activity	Intracellular membranes
CD163L1	Nucleosome/cytosol/centrosome	Spleen (Enhanced)	Homologs co-express with VTCN1^‡^°	Signaling receptor binding	PM/cell junction
NKG7	Vesicles	Spleen/bone marrow (Both enhanced)	GZMA/B/H/K° PRF1°	Apoptosis and cell death	Secreted proteins
TMEM254	PM/nucleoplasm	Expressed-in-all	–	(Conserved Pfam domain of unknown function: PF14934)	–
MYEOV	Nucleus/vesicles	–	CNN1°	Smooth muscle contraction	Actin filaments
TMEM160	–	Mixed	SAC3D1	Cell division	Cytosol
ITPRIPL2	Centrosome	Expressed-in-all	MYH6	Muscle contraction	Cytosol

The uncharacterized proteins are the first column while the interacting proteins (fourth column) with bold font and italics are members of the 3TM dataset. Interacting/co-expressing proteins have low^∧^ or medium confidence scores based on co-expression° or experimental evidence* or text mining from literature^‡^ from String protein-protein interaction networks (Szklarczyk et al., 2015) or from IntAct molecular interaction database^!^ (Orchard et al., 2014). Protein localization information obtained from gene ontology (GO) Annotation (Huntley et al., 2015) and cell atlas (Thul et al., 2017). The top three interactions were selected and interactions with other 3TM proteins in the dataset have been preferentially selected. MVB, multivesicular bodies; ER, endoplasmic reticulum; PM, plasma membrane.

Intriguingly, the three uncharacterized proteins TMEM235, TMEM151A, and TMEM151B exhibit enriched tissue-expression in the cerebral cortex and all are identified in String interaction networks (Szklarczyk et al., 2015) to co-express with MAG, an adhesion molecule that is a myelin-associated glycoprotein, and also with OPALIN, which is the oligodendrocytic myelin paranodal and inner loop protein. TMEM151A and TMEM151B, which both contain a conserved but uncharacterized Pfam domain (PF14857), co-express with TMEM235, but not with each other. TMEM235, as well as TMEM114 which is another uncharacterized 3TM protein, both contain the Claudin_2 conserved Pfam domain (PF13903) and were shown to resolve within the distantly related voltage dependent calcium channel gamma subunits (CACNGs) rather than within the 27 members of the human Claudin family. However, the functional activities of these uncharacterized proteins have yet to be determined, although it is interesting to speculate that with the conserved claudin domain and co-expression with other adhesion and myelin associated proteins, this trio of uncharacterized proteins may be involved as structural components of oligodendrocytic cells or loops.

Piecing together the diverse information on the uncharacterized protein TMEM178B reveals a potentially important protein that exhibits enhanced expression in the cerebral cortex and localizes to vesicles and the nucleoli. It also contains the conserved Claudin_2 Pfam domain (PF13903), of which family members perform diverse functions. In a recent analysis that produced a protein-protein interaction map of the TNF-induced NF-κB signal transduction pathway, TMEM178B was found to interact with two proteins, TNIP2 and TRAF2, which both inhibit or regulate NF-κB signaling and kinase activity and are also involved in apoptotic functions (Van Quickelberghe et al., 2018). Furthermore, TMEM178B-BRAF gene fusions were identified in two primary mucosal malignant melanoma cases, which are more aggressive than cutaneous melanomas (Kim et al., 2017). And another recent study concluded that oncogenic BRAF fusions may be targeted therapeutically by the combination of a MEK inhibitor with a PI3K or CDK4/6 inhibitor (Kim et al., 2017). Hence, while the exact functional activities of TMEM178B remain unknown, results suggest that it may be oncogenic and involved in disease progression, and we might infer that it could be associated with important signaling pathways.

## Materials and Methods

### Homo Sapiens Proteome Retrieval

The *Homo sapiens* current protein sequences (*CCDS_protein.current.faa*) using the GRCh38.p7 assembly with consensus coding sequence (CCDS) (Pruitt et al., 2009) annotation was downloaded from the National Center for Biotechnology Information (NCBI) website (National Center for Biotechnology Information, 2018). The following files were also downloaded from this website: *CCDS2UniProtKB.current.txt*, which gives the UniProtKB identifier corresponding to each CCDS identifier; *CCDS_attributes.current.txt*, which shows the corresponding gene and various attributes for each CCDS identifier; *CCDS2Sequence.current.txt*, with information on the nucleotide and protein ID and status in CCDS; and *CCDS.current.current.txt*, specifying more on CCDS status along with other information. CCDS is a collaborative effort to produce consensus annotation of a standard set of human genes that also includes alternative splicing sequences. The annotation uses manual curation through the Havana (Havana Annotation by Wellcome Sanger Institute, 2018) and RefSeq groups as well as automatic methods from ENSEMBL (Ensembl genome browser 91, 2018) and NCBI computational pipelines.

### Transmembrane Prediction

Transmembrane prediction algorithms have difficulty differentiating between N-terminal alpha-helices and cleavable signal peptides. Thus, the protein sequences were assessed using SignalP v4.1 on an installment on the UPPMAX high-performance computing service. The default parameters were used with eukaryotic type of organism and the *best method* was selected which indicated transmembrane regions might be present. The mature sequences with the signal peptides excised were collated and evaluated with TOPCONS-single (Hennerdal and Elofsson, 2011) transmembrane prediction web server to assess membrane topology, including the number of membrane-spanning helices and the orientation of the N- and C-terminals. Prediction methods use different algorithms to discriminate transmembrane helices which results in possibly different numbers of helices within a protein. To improve the accuracy of transmembrane prediction methods, a consensus or majority decision using several different algorithms is preferred. TOPCONS-single, which is suitable to use for large proteome datasets, is a consensus method that incorporates multiple methods and uses a hidden-Markov model to estimate the consensus topology for a predicted transmembrane protein. The default methods used were SCAMPI-single (Bernsel et al., 2008), S-TMHMM (Viklund and Elofsson, 2004), HMMTOP (Tusnády and Simon, 2001), and MEMSAT (Jones et al., 1994). The resulting proteins identified as 2TM 3TM, and 4TM were retrieved. This included canonical sequences as well as isoforms. In early 2019 SignalP v5.0 was released that improved signal peptide predictions by using deep neural networks (Armenteros et al., 2019b) and the original sequence versions of the 2TM, 3TM, and 4TM groups of proteins were re-evaluated using SignalP v5.0 and then assessed with TOPCONS2.0. This transmembrane prediction software is also a consensus membrane prediction server, however, it is a more recent iteration of the TOPCONS series and can more successfully predict membrane topologies (Tsirigos et al., 2015). Furthermore, TOPCONS2.0 was chosen due to the robust benchmark sets used in assessing the software which posited ∼80% accuracy in predicting transmembrane proteins (Tsirigos et al., 2015), whereas other resources used smaller benchmark sets to state possibly higher accuracy. As reliability is still an issue for any membrane prediction resource, we further attempted to assess questionable proteins and protein families in the dataset through corroboration with other transmembrane prediction resources such as the Human Transmembrane Proteome database (Dobson et al., 2015), and also the human protein atlas which also uses a majority consensus method to determine signal peptides and transmembrane topology (Fagerberg et al., 2010). Additionally, comparisons of specific proteins or domains to experimentally determined 3D structures were evaluated. SignalP v5.0, SignalP v4.1, and TOPCONS2 were used to assess the presence of signal peptides and N-terminal topology. Several localization prediction resources were used to asses predicted versus annotated localization regions. ERPred uses split amino acid composition as support vector machine input to predict ER resident proteins (Kumar et al., 2017). DeepLoc uses sequence information with deep neural networks to predict subcellular localization of proteins (Almagro Armenteros et al., 2017). TargetP-2.0 uses deep learning to predict N-terminal signal peptides and mitochondrial transit peptides among others (Armenteros et al., 2019a). The predicted 3TM dataset was assessed for canonical or alternative splicing sequences using the UniProt identifiers (obtained from *CCDS2UniProtKB.current.txt*), where isoforms typically have an additional number at the end of the identifier. The canonical sequence is determined as the most prevalent, the most similar to orthologous sequences, the amino acid properties in the sequence, or else the longest sequence.

### Protein Annotation and Information

Universal protein resource, UniProt, is a central repository for protein annotation data with both manually curated and automatically analyzed information (Consortium, 2015). Protein annotations for the canonical 3TM proteins were obtained from the website and included: review status, transporter classification number, EC, GO annotation terms, and protein family information. The 3TM proteins were also searched against the Pfam (Finn et al., 2016) database (v31) using an installment on the UPPMAX high-performance computing service. Pfam is a collection of protein families and domains, and also higher-level groupings of related entries called clans, and are represented by multiple sequence alignments and hidden Markov models. The IUPHAR/BPS Guide to Pharmacology (Harding et al., 2018) *targets_and_families.csv* file was downloaded and evaluated to aid in functional classifications of the dataset.

The Jensen lab DISEASES database (Pletscher-Frankild et al., 2014) is a resource that integrates evidence by assigning confidence scores on disease-gene associations from automatic text mining, manually curated literature, cancer mutation data, and genome-wide association studies. The *human_disease_knowledge_filtered.tsv* file was downloaded from their website and evaluated for gene-disease associations with the 3TM dataset. Additionally the DisGeNET drug encyclopedia was also utilized to assess genes associated to human diseases (Piñero et al., 2017). The evidence metrics *limited*, *moderate*, and *strong* were used to identify relevant associations. To identify possible drug targets in the dataset, the DrugBank annotations that were obtained via UniProt were further investigated (Wishart et al., 2017). Furthermore, an updated dataset from Rask-Andersen et al. (2014) that provides curated information for all current targeted as well as investigative proteins in clinical trials was obtained (Attwood et al., 2018).

The Kyoto Encyclopedia of Genes and Genomes, KEGG, is a database resource that assigns functional meaning to genes and genomes at molecular and higher levels (Kanehisa et al., 2017). BlastKOALA, which is the KEGG webserver for automatic annotation of query sequences, was used to obtain information on the pathways, or molecular interactions and relations, for networks that included: metabolism, genetic information processing, environmental information processing, cellular processes, organismal systems, human diseases, and drug development.

### Gene Enrichment Analyses: Gene Ontology Annotation and TissueEnrich Analyses

The PANTHER Classification System (version 14.1; released July 11, 2019) (Mi et al., 2019) overrepresentation test was used to analyze gene enrichment in the 3TM dataset in comparison to the entire human membrane proteome. PANTHER is a comprehensive resource for the functional classification of genes using the GO annotations (released July 03, 2019). The PANTHER Overrepresentation Test uses the Fisher’s exact test, which assumes a hypergeometric distribution that is more accurate for smaller gene lists, and also uses the Benjamini-Hochberg False Discovery Rate (FDR) correction (*p* < 0.05) to control the false positive rate in the statistical test results (Mi et al., 2019). The annotation data sets included PANTHER GO-Slim Molecular Function, PANTHER GO-Slim Biological Process, and PANTHER GO-Slim Cellular Component, as well as the GO complete sets. The Reactome pathways (version 65; released March 12, 2019) data set and also PANTHER protein classes (version 14.1; released March 12, 2019) were also used. QuickGO annotations were also applied for additional protein annotation (Binns et al., 2009; Huntley et al., 2015). The reference protein list for the *homo sapiens* membrane proteome was obtained from (Attwood et al., 2017).

The recently released website resource TissueEnrich: Tool for tissue-specific gene enrichment in human and mouse (Jain and Tuteja, 2019) was used to analyze tissue-specific gene enrichment in the 3TM dataset in comparison to the human membrane proteome. TissueEnrich defines tissue-specific genes using RNA-Seq data from the human protein atlas, GTEx, and mouse ENCODE data sets. Tissue-specific genes are defined as: Not Expressed: Genes with an expression level less than 1 (TPM or FPKM) across all the tissues; Tissue Enriched: Genes with an expression level greater than or equal to 1 (TPM or FPKM) that also have at least five-fold higher expression levels in a particular tissue compared to all other tissues; Group Enriched: Genes with an expression level greater than or equal to 1 (TPM or FPKM) that also have at least five-fold higher expression levels in a group of 2–7 tissues compared to all other tissues, and that are not considered Tissue Enriched; and Tissue Enhanced: Genes with an expression level greater than or equal to 1 (TPM or FPKM) that also have at least five-fold higher expression levels in a particular tissue compared to the average levels in all other tissues, and that are not considered Tissue Enriched or Group Enriched. TissueEnrich uses the hypergeometric test to calculate the enrichment of tissue-specific genes in the 3TM data set and the Benjamini-Hochberg correction for multiple hypotheses (Jain and Tuteja, 2019). The parameter P-adjusted was selected in the Histogram Plot Options. The human protein atlas dataset was used in the enrichment tests.

### Uncharacterized Proteins

The uncharacterized proteins were investigated further using the Cell Atlas (Thul et al., 2017) to obtain subcellular localizations where possible. String Protein-Protein Interaction Networks (Szklarczyk et al., 2015) and IntAct Molecular Interaction Database (Orchard et al., 2014) were utilized to retrieve interacting proteins with emphasis selected for other 3TM proteins within the database. The functions of the interacting proteins were obtained from UniProt (Consortium, 2015).

All analysis and classifications were performed using local Python and Perl script and SQL databases (sqlite3). Adobe Illustrator CS6 was used for the figures.

## Conclusion

This analysis characterizes the structurally similar 3TM group and assimilates information on statistically relevant functional activities along with cellular localizations, tissue enrichment patterns, and protein-protein interaction networks to describe the prevailing functional activities of this group of proteins. Trispanins contain many evolutionarily conserved proteins that are predominantly localized to intracellular organelles and specifically to the ER. This group of proteins is primarily involved in aspects of cellular membrane composition and trafficking systems including membrane synthesis, protein trafficking, structural components, and are members of important membrane complexes. Further, the 3TM dataset contains the large and important ionotropic glutamate receptor superfamily which is involved in fast signal transduction in the brain. The methodology employed in this study uses bioinformatic analyses to identify uncharacterized proteins potentially involved in significant activities or disease pathways and provides means to reasonably speculate on the functional activities of several intriguing uncharacterized proteins.

## Data Availability Statement

All datasets generated for this study are included in the article/Supplementary Material.

## Author Contributions

MA contributed in conceiving the project, created the dataset, analyzed the data, and drafted the manuscript. HS contributed in conceiving the project and editing the manuscript.

## Conflict of Interest

The authors declare that the research was conducted in the absence of any commercial or financial relationships that could be construed as a potential conflict of interest.

## Supplementary Material

MATERIAL S1 The 3TM dataset is presented in Excel format in the file Trispanins Dataset. The details of the statistical results from the PANTHER overrepresentational analysis are in Supplementary Table.
